# Lifetime prevalence of polyvictimization among older adults in Sweden, associations with ill-heath, and the mediating effect of sense of coherence

**DOI:** 10.1186/s12877-021-02074-4

**Published:** 2021-02-17

**Authors:** Johanna Simmons, Katarina Swahnberg

**Affiliations:** 1 Department of Acute Internal Medicine and Geriatrics in Linköping, and Department of Health, Medicine and Caring Sciences, Linköping University, Linköping, Sweden; 2 Department of Health and Caring Sciences, Faculty of Health and Life Sciences, Linnaeus University, Kalmar, Sweden

**Keywords:** Polyvictimization, Violence, Abuse, Life-course perspective, Resilience, Mediation model, PROCESS, Elder Abuse

## Abstract

**Background:**

Experiences of violence and abuse is a prominent part of the life history of many older adults and is known to have negative health effects. However, the importance of multiple victimization over the life course, e.g., lifetime polyvictimization, is not well investigated in this age group. The objective of this study was to investigate the prevalence of lifetime physical, emotional, and sexual victimization as well as polyvictimization among older adults in Sweden. We explored background characteristics associated with polyvictimization and hypothesized that violence victimization and especially polyvictimization would be associated with lower health status. To better understand factors that promote health in the aftermath of victimization, we also explored the effect of two resilience factors, sense of coherence (SOC) and social support, on the association between victimization and ill-health.

**Method:**

Cross-sectional data from a random population sample in Sweden (women *n* = 270, men *n* = 337) aged 60–85 was used. Respondents answered questions about exposure to violence, health status, social support, and SOC. Conditional process analysis was used to test if SOC mediates the association between victimization and health outcome, and if social support moderates the association.

**Results:**

Overall, 24.8% of the women and 27.6% of the men reported some form of lifetime victimization and 82.1% of the female and 62.4% of the male victims were classified as polyvictims, i.e., reported experiences of more than one episode of violence. As hypothesized, we found a negative association between victimization and health status and the association was most prominent for polyvictims. We found moderated mediation for the association between polyvictimization and health status, i.e., polyvictimization was associated with lower SOC and SOC had a positive correlation with health status. Social support moderated the association, i.e., victims without social support had lower health scores.

**Conclusions:**

Lifetime polyvictimization was common among older adults and associated with lower health status. To help victims of violence recover, or preferably never develop ill-health, a better understanding of what fosters resilience is warranted. This study implies that social support, and especially SOC may be factors to consider in future interventions concerning older adults subjected to violence.

## Background

Experiences of violence and abuse is a prominent part of the life history of many older adults, but the importance of multiple victimization over the life course, e.g., lifetime polyvictimization, is not well investigated in this age group [1, 2]. Although different forms of victimization are associated with poor health outcome, not all victims develop ill-health in the aftermath of violence and a better understanding of what fosters resilience is warranted [2]. The objective of this study therefore was to investigate the prevalence of lifetime experiences of physical, sexual, and emotional violence as well as lifetime polyvictimization in different relational contexts (i.e., by an intimate partner, a family member, or other person) among older adults in Sweden. Also, we aimed to explore the effect of two resilience factors, sense of coherence (SOC) and social support, on the expected association between victimization and physical and mental ill-health.

### Polyvictimization

One of the strongest risk factors for being subjected to violence is previous victimization [3–6]. For example, associations between childhood abuse and intimate partner violence in adulthood are well-known. Recent findings have also shown a correlation between reporting childhood abuse and experiencing elder abuse [7, 8]. Polyvictimization was first introduced as a concept among children and youth, showing that the total number of types of victimization is a better predictor of both mental and physical ill-health than any one form of violence alone [6, 9, 10]. This is consistent with research on trauma and post-traumatic stress disorder (PTSD), suggesting that prior trauma, even merely stressful life events, has a sensitizing effect on victims, increasing the risk of developing PTSD in the aftermath of a new trauma [11–14]. In addition, traumatic life events may have long standing health effects, e.g., childhood trauma has been associated with poor health outcome among older adults [15, 16]. The intertwined nature and chronic health effects of victimization underline the need for a life-course perspective in research on interpersonal violence. Such a perspective is also consistent with the theory of cumulative inequality in gerontology, emphasizing that events and experiences earlier in life shape later life outcomes [17]. Theory of cumulative inequality builds on theories of cumulative advantage and disadvantages, stipulating that inequality systematically cumulates and shapes life trajectories [18]. Applied to violence research, polyvictimization could potentially be understood as a form of cumulative disadvantage where prior victimization increases the odds of renewed victimization as well as leads to cumulative adverse health effects.

As for children and youth, polyvictimization among older adults may be a more important predictor of ill-health and repeated victimization than any one form of violence alone. If that is the case, this would likely affect policy programs for detecting and managing elder abuse. The polyvictimization framework is currently being applied in more and more studies and its importance is also increasingly acknowledged in research concerning older adults, albeit only rarely investigated [1, 2, 15, 19, 20]. Elder polyvictimization can be defined as when *“a person aged 60 or older is harmed through multiple co-occurring or sequential types of elder abuse by one or more perpetrators or when an older adult experiences one form of abuse perpetrated by multiple others with whom the older adult has a personal, professional, or care recipient relationship in which there is a societal expectation of trust”* [20]. Williams et al. [19] found that compared to non-victims and victims reporting one form of elder abuse in the past year, those that reported past year elder polyvictimization were more likely to report poor health in bivariate but not multivariate analysis. Worse health outcome for victims of repeated victimization in later life have been found previously, without being framed as polyvictimization [21, 22]. In this study, all respondents reporting more than one violent incident are classified as polyvictims. However, because we have a life-course perspective, victimization is not limited to relationships with an expectation of trust but includes all kinds of perpetrators, e.g., partners, other family members, acquaintances, and strangers.

### Life history of violence among older adults in Sweden

To appreciate the cumulative burden of victimization for older adults, studies should consider polyvictimization across the lifespan [2]. Only a few studies have investigated violence victimization among older adults in Sweden, and none of them have considered lifetime polyvictimization. However, prevalence of victimization in the past year among older adults has been investigated [23–25], as well as prevalence of different kinds of victimization since the age of 18 [26]. Ahnlund et al. [23] report that 6% of women and 3% of men, age 60–74, have experienced at least one episode of physical, sexual, or emotional violence by any type of perpetrator during the latest 12 months. Using the same sample, lifetime prevalence of repeated emotional violence, but not physical or sexual violence, was reported in one population study and found to be 25% for both male and female respondents, age 55–74 [27]. Another study reported the prevalence of elder abuse (sexual, physical, emotional, economic, and neglect) since the age of 65 and found it to be 16% among older women and 13% among older men in Sweden [28].

### Background characteristics associated with polyvictimization

Subjective position and resources have been argued to be more important than actual ones in shaping life trajectories [17]. Therefore, in this study, we use a subjective measure of social status that previously has been found to be strongly correlated with ill-health. The relationship could only partly be accounted for by objective measures of social status, such as education, occupation, and income [29, 30]. In the American National Elder Mistreatment Study, factors that might indicate low social status, e.g., lower income, unemployment, belonging to a racial minority, and use of social services, were related to reporting elder polyvictimization. A positive correlation was also found between being married or cohabiting and reporting elder polyvictimization, while the educational level was unrelated. Older age was negatively correlated with reporting elder polyvictimization [19].

### Resilience factors

Not all victims of violence develop ill-health. There is a growing interest in research on factors that promote resilience among victims [2, 15, 16, 31–33]. A better understanding of what protects victims from ill-health in the aftermath of violence might potentially be used in intervention programs for victims so that the negative health effects of victimization can be limited. In this study, we will investigate two resilience factors: social support and ‘sense of coherence’ (SOC). The latter was introduced by Aaron Antonovsky as salutogenic factors within a person, helping them to deal with difficult life events, e.g., victimization. SOC is comprised of a sense of comprehensibility, manageability, and a feeling that life is meaningful [34]. SOC is positively correlated with good health [35] and negatively correlated with symptoms of PTSD [36]. Also, SOC has been found to have a mediating effect on the association between violence at the workplace and stress symptoms, i.e., victimization was associated with a lower SOC, which, in turn, was associated with more stress symptoms [37]. For polyvictims, compared to those reporting one form of victimization, more relationships and environments are affected by violence. As a consequence, more traumatic reminders may be present that interfere with normal coping [6]. It is therefore possible that the association between SOC and victimization is different for those reporting only one form of victimization compared to polyvictims.

Originally, SOC was thought of as a static personality trait, but evidence suggest that it is not as stable over time as first thought [38]. For example, one study concerning factors associated with staying well or burning out at work included a 10 year follow up in which participants’ SOC was reported to have changed. Respondents reporting serious burnout reported a negative change in SOC over the 10 years while respondents with no burnout reported a positive change [39]. It has also been reported that the mean score of SOC tends to be higher in older samples indicating that SOC increase with age [38].

Low social support has repeatedly been reported as a risk factor for elder abuse, including elder polyvictimization [19, 23, 40, 41]. Strong social support, on the other hand, is well known to be correlated positively with health status [42]. One study from Mexico found that social support moderates the association between elder abuse and depression among older women [43], and supportive interpersonal relationships have been considered to be a resilience factor after polyvictimization among adolescents and adults [32].

### Aim and hypotheses

Taken together, there is a need for more studies investigating polyvictimization among older adults from a life-course perspective and including both measures of ill-health and resilience factors [2, 16]. Therefor the aims of this study were as follows:

1. Explore lifetime prevalence of experiencing sexual, physical, and emotional violence as well as polyvictimization, among older men and women (age 60–85) in Sweden.
2. Explore associations between background characteristics and polyvictimization. We hypothesized that younger age, female sex, being married, low subjective social status, and low social support would be associated with increased odds of reporting polyvictimization.
3. Explore how the association between victimization and mental as well as physical health status is influenced by SOC and social support. We propose a model stipulating
  - A direct negative effect of victimization on health status and that the effect is more prominent for polyvictims than for those reporting only one form of victimization.
  - An indirect negative effect of victimization on health status, mediated by a SOC, i.e., victimization is associated with lower SOC and lower SOC is in turn associated with lower health status.
  - Social support moderates both the direct and the indirect effect of victimization on health status, with social support being associated with better health status.

## Method

### Population and procedure

This study is a cross-sectional study and data consist of a sub sample of older adults (age 60–85) taken from a previous population study including adults, age 25–85, in Sweden. The study was conducted between September and November 2012 [44] and the focus of the original study was experiencers of violence, ill-health and health care response to victims. For the original study, the population register was used to sample 2200 men and 2000 women, age 25–85, at random from a county in southeastern Sweden. An information letter about the study was sent to participants together with a questionnaire (described under the heading “measurement”). Hence, respondents were approached by postal mail but were given the opportunity to answer by paper or online. Two reminders were sent by postal mail. No incentives were given for participation. A filled in questionnaire, either on paper or online, was considered as informed consent to participate in the study. The response rate was 35% among men and 38% among women.

In this study, all respondents aged 60–85 years were included. Four respondents, who did not answer any of the questions concerning experiences of violence, were excluded, leaving a total sample of 270 women and 337 men. Background characteristics of participants are presented in Table 1. A thorough examination of the non-response bias has been published previously [44]. Essentially, when adjusted for differences in background characteristics, no difference was found for prevalence of violence regardless of the mode of response, i.e., paper or online. Likewise, there was no difference in prevalence regardless of whether the response was given promptly or after one or two reminders.

**Table 1:** Background characteristics of the participants

		Women		Men	
		N	%	N	%
**Age**	60–64	77	28.5	82	24.3
	65–69	81	30.0	97	28.8
	70–74	60	22.2	71	21.1
	75–79	34	12.6	53	15.7
	80–85	18	6.7	34	10.1
**Civil state**	Single	68	25.2	55	16.7
	Partner	202	74.8	280	83.32
**Subjective social status (score)**	1–4	62	25.3	64	20.2
	5–7	146	59.6	201	63.4
	8–10	37	15.1	52	16.4
**Social support**	No	31	12.1	52	16.0
	Yes	226	87.9	273	84.0

Note: “Partner” = Married, co-habiting, steady relationship

Posing questions about violence can be a sensitive topic, triggering memories and flashbacks for victims. Contact information to an independent therapist was therefore provided in the letter accompanying the questionnaire. The study was approved by the regional ethical review board in Linköping, Sweden (register no 2012/194–31).

### Measurements

The questionnaire used to collect data contained several instruments. The measurements used in the present study have been used repeatedly in different studies and validity measures are described further on for each specific instrument.

#### Life history of violence

Life history of violence was measured using the NorVold Abuse Questionnaire (NorAQ), including questions about emotional, sexual, and physical violence. NorAQ has been validated in Swedish male and female samples, using an interview as the gold standard [45, 46]. The validity measures found were satisfactory. For emotional violence: women: sensitivity 75%, specificity 98%, positive likelihood ratio (LR) 38; men: sensitivity 83%, specificity 72%, LR 3. For sexual violence: women: sensitivity 83%, specificity 98%, LR 42; men: sensitivity 68%, specificity 99%, LR 46. For physical violence: women: sensitivity 96%, specificity 85%, LR 6; men: sensitivity 76%, specificity 92%, LR 9 [45, 46]. Because the question about mild physical violence in NorAQ had comparably low concurrent validity in the validation studies, it was removed from this study. Hence, only two items cover physical violence. Items concerning exposure to violence are presented together with prevalence rates in Table 2. A comparison of NorAQ to other similar instruments have been published previously [44].

**Table 2:** Prevalence of lifetime violence victimization (women *n* = 270, men *n* = 337)

	Women		Men		*p*-value
	N	%	N	%	
Any violence	67	24.8	93	27.6	0.44
One form of victimization	12	**4.4**	35	**10.4**	**0.01**
Polyvictimization	55	20.4	58	17.2	0.31
**Emotional violence**					
Systematically repressed, degraded, humiliated	28	10.4	32	9.5	0.71
Limited contact with others, controlled	17	6.3	19	5.6	0.72
Living in fear because of threats	19	7.0	15	4.5	0.18
Any emotional violence	35	13.1	41	12.4	0.80
**Physical violence**					
Hit with fist, hard object, kicked, pushed violently, beaten, thrashed, or similar	43	15.9	60	17.8	0.54
Life-threatened, by e.g., trying to strangle you, showing a weapon/knife, or similar	12	4.4	27	8.0	0.07
Any Physical violence	47	17.7	72	22.2	0.17
**Sexual violence**					
Sexual humiliation	**7**	**2.6**	**2**	**0.6**	**0.04**
Touched body parts other than genitals	25	9.3	5	1.5	**< 0.01**
Touched or forced to touch genitals, used your body to satisfy him/herself sexually	26	9.6	6	1.8	**< 0.01**
Put or tried to put penis or object in vagina, mouth, or rectum	16	5.9	0	0	**< 0.01**
Any sexual violence	40	**15.1**	7	**2.2**	**< 0.01**
**Kind of perpetrator**					
Family	24	8.9	22	6.5	0.27
Partner	33	**12.2**	14	**4.2**	**< 0.01**
Other	43	**15.9**	78	**23.1**	**0.03**
**Characteristics of victimization (only victims)**					
One form of victimization	12	**17.9**	35	**37.6**	**0.01**
Low polyvictimization	38	**56.7**	37	**39.8**	**0.04**
High polyvictimization	17	25.4	21	22.6	0.68
Two or more forms of violence	42	**62.7**	24	**25.8**	**< 0.01**
Two or more perpetrators	27	**40.9**	21	**23.6**	**0.02**

##### Polyvictimization

We wanted to capture the overall burden of lifetime experiences of violence and create a measure of polyvictimization. Therefore, for this study, the response alternatives in NorAQ were slightly modified compared to the original version used in the validation studies. Each question concerning violence was first answered with a yes or no. Those answering yes were then asked to specify who the perpetrator was (family, partner, other). For each category of perpetrator, the frequency (sexual and physical violence: 1–2, 3–5, 6–9, > 10 times) or duration (emotional violence: < 6 months, 6–12 months, 1–2 years, > 2 years) of victimization was selected. For each perpetrator, on each individual question, a score between 0 (no violence) and 4 (> 10 times/> 2 years of) was given. To capture the overall burden of victimization, the score for each question was than computed into a sum score (theoretical range 0–108, reported range 0–44). For example, someone reporting mild emotional violence by a partner during 6–12 months (score = 2), mild emotional violence during more than 2 years by a family member (score = 4), and moderate sexual violence on 1–2 occasions by a partner (score = 1) would have a sum score = 7. Hence, the sum score increases as the number of forms of violence, kinds of perpetrators as well as duration/frequency of violence increase. Five respondents only responded to the first yes/no question and not the following questions concerning perpetrator, duration, or frequency. They were given the score “1” for each question they answered affirmatively.

In the original polyvictimization study, a cut-off score was chosen for high polyvictimization so that approximately one-third of the polyvictims were classified as high polyvictims [6]. Based on the same principle, we used our sum score of victimization to categorize respondents as non-victims (0 point: *n* = 447, 73.6%), victims reporting one form of violence (1 point: *n* = 47, 7.7%), low polyvictims (2–7 points: *n* = 75, 12.7% of all 66.4% of polyvictims), and high polyvictims (8 or more: *n* = 38, 6.3% of all, 33.6% of polyvictims).

#### Ill-health

We used the Swedish version of the 12-item short form survey (SF-12) to measure ill-health [47]. SF-12 is a generic measure that does not target a specific age or disease; rather, it is used to measure general health and well-being from the respondents’ perspectives. Items cover respondent’s perception of health-related difficulties e.g., to what extent the respondents experience that their physical or mental health limits their ability to perform everyday work and activities. Respondents’ mental state is also covered, e.g., feeling calm and harmonious, feeling energetic, and feeling gloomy and sad. The principal component analysis of the correlation between the items has revealed two underlying constructs of the instrument, i.e., the Mental Component Summary (MCS) and the Physical Component Summary (PCS) subscales, which are interpreted as physical and mental components of health status. Using a principal component analysis, we were able to confirm the intended two factor solution in this sample, explaining 71% of the variance. Cronbach’s alpha for this sample was 0.92 for PCS and 0.88 for MCS. Responses were therefore scored, weighted, and transformed, according to the manual to create the MCS and PCS subscales [48].

#### Sense of coherence

To measure SOC we used the short version of Antonovsky’s SOC instrument [34, 38]. It consists of 13 items covering three domains: 1) Comprehensibility, five items, e.g., Has it happened in the past that you were surprised by the behavior of people whom you thought you knew very well? 2) Manageability, four items, e.g., Do you have the feeling that you’re being treated unfairly? and 3) Feeling that life is meaningful, four items e.g., How often do you have a feeling that there’s little meaning in the things you do in your daily life? Respondents ranked their response on each item on a seven-point scale, and a sum score was created (range 13–91), where a higher score indicates better SOC. If one or two items had missing data, this was replaced by the mean; if three or more items had missing data, the respondent was excluded from the analyses using the SOC variable. The SOC scale has previously been found to be a reliable, valid, and cross culturally applicable instrument [38]. Cronbach’s alpha in this sample was 0.72.

#### Social support

One item derived from the annual Swedish national public health survey [49] was used to measure social support: Do you have anyone you can share your innermost feelings with and confide in? Possible answers were yes or no.

#### Subjective social status

We used a measure of subjective social status that consists of a drawing of a ladder with 10 rungs [29, 30]. The following instruction was found next to the ladder: “Think of this ladder as representing where people stand in our society. At the top of the ladder are the people who are best off, those who have the most money, most education, and the best jobs. At the bottom are the people who are the worst off, those who have the least money, least education, and the worst jobs.” Respondents were asked to place an “X” on the rung that best represented where they think they stand on the ladder [29, 30].

### Statistics

#### Power calculation

Our original sample size calculation is not relevant to this study as it concerns the whole sample, including all age groups. A power calculation for this specific subsample of the original data set was calculated using an online tool, Openepi.com [50]. As our primary focus was on the association between victimization and ill-health, we calculated the power for comparing the means on the MCS and PCS between non-victims (*n* = 447) and polyvictims (*n* = 113). The proposed minimal important difference for the MCS and PCS (three T-score points) was used together with the standard deviation found in the Swedish validation of SF-12 (MCS = 9.6, PCS 8.5) [48, 51]. This generated a power of 84.3% for detecting differences in MCS score and 91.8% for detecting differences in the PCS score.

#### Statistical analysis

In all analysis, the significance level was set to 95%. Unless otherwise noted IBM SPSS statistics version 25 was used for statistical analyses.

**For aim 1,** descriptive statistics were used to investigate a) the prevalence of the different forms of violence, b) proportion of victims reporting more than one form of violence, c) proportion of victims reporting more than one kind of perpetrator, and d) categorize respondents as non-victims, victims reporting one form of violence, low polyvictims, and high polyvictims. Differences in the prevalence of violence between the sexes were analyzed using chi-square test in MedCalc for Windows, version 15.0 [52] (Table 2).

**For aim 2,** multinomial regression analysis was used to test if the background characteristics (age, sex, civil status, subjective social status, social support) were associated with reporting victimization (non-victims, single, low polyvictims, high polyvictims) (Table 3).

**Table 3:** Association between background characteristics and reporting polyvictimization

		One form of victimization (*n* = 44, 8.2%)			Low polyvictimization (*n* = 66, 12.2%)			High polyvictimization (*n* = 34, 6.3%)		
		Exp (B)	95% CI		Exp (B)	95% CI		Exp (B)	95% CI	
**Age**	60–85	0.97	0.92	1.03	0.97	0.93	1.01	0.96	0.90	1.02
**Sex**	Female	1			1			1		
	Male	**2.25**	**1.10**	**4.61**	0.93	0.55	1.59	1.09	0.52	2.27
**Civil state**	Single	**3.39**	**1.60**	**7.20**	1.60	0.82	3.14	1.38	0.57	3.32
	Partner	1			1			1		
**Subjective** **social status**	1–10	1.06	0.89	1.26	0.97	0.84	1.12	0.90	0.75	1.09
**Social support**	No	2.15	0.94	4.93	1.60	0.74	3.45	**4.77**	**2.08**	**10.94**
	Yes	1			1			1		

Note: Multinomial regression was used, and reference category is “no victimization” (*n* = 395). Included in analysis 539, missing 68, total 607. Model fit Cox & Snell R square 0.08, Nagelkerke 0.10. “Partner” = Married, co-habiting, steady relationship

**Fig. 1:**
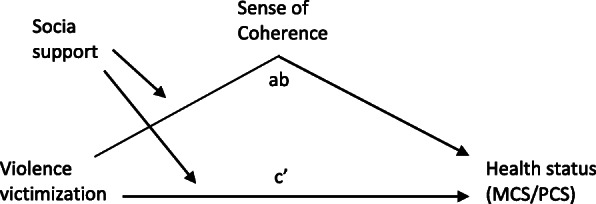
Our proposed model of association between violence victimization and health status. *Victimization was hypothesized to have both a direct effect* (c’) *and an indirect effect (ab, mediation through SOC) on health status. Social support was hypothesized to moderate the associations*

**For aim 3,** conditional process analysis was used to test our proposed model. This type of analysis can be used to understand and describe mechanisms by which a variable affects other variables [53]. We hypothesized that victimization had both a direct effect and an indirect effect mediated by SOC on health status, and that social support moderated the associations. Our proposed model of the association is illustrated in the Fig. 1. Two models were created, one with the PCS and one with the MCS as the outcome measure. The models were tested using the PROCESS tool, which is an SPSS macro for examining mediations models, based on regression analyses [53]. We used 5000 bootstrap samples to calculate 95% confidence interval (CI). Violence victimization was considered a multicategory variable, and “no violence” was set as the reference category, meaning that all estimates given are for comparison with no victimization. The models were adjusted for age, sex, civil status, and subjective social status.

## Results

Background characteristics of the sample are presented in Table 1.

### Aim 1

The prevalence of victimization is reported in Table 2. Overall, 24.8% (95% CI 19.8–30.4%) of the women and 27.6% (95% CI 22.9–32.7%) of the men reported some form of victimization. More women than men reported sexual violence (women 15.1%, men 2.2%, *p* < 0.01) and violence by a partner (women 12.2%, men 4.2%, *p* < 0.01). More men than women reported “other” as the perpetrator (women 15.9%, men 23.1%, *p* = 0.03). Polyvictimization was reported by 20.4% (95% CI 16.0–26.0%) of the women and 17.2% (95% CI 13.3–21.7%) of the men in the total sample, which constitutes 82.1% of female and 62.4% of male victims. One form of victimization was more common among male victims (women 17.9%, men 37.6%, *p* < 0.01), while low polyvictimization was more common among female victims (women 56.7%, men 39.8%, *p* = 0.04). High polyvictimization was evenly distributed among male and female victims (women 25.4%, men 22.6%, *p* = 0.68). More female than male victims reported two or more forms of violence (i.e., emotional, physical, sexual) (women 62.7%, men 25.8%, *p* < 0.01) as well as two or more kinds of perpetrators (i.e., family, partner, other) (women 40.9%, men 23.6%, *p* = 0.02).

### Aim 2

Being single (OR 3.39, 95% CI 1.6–7.2) and being male (OR 2.25, 95% CI 1.1–46) were associated with reporting one form of victimization. The only background characteristic associated with reporting high polyvictimization was low social support (OR 4.77, 95% CI 2.08–10.94) (Table 3).

### Aim 3

**Fig. 2:**
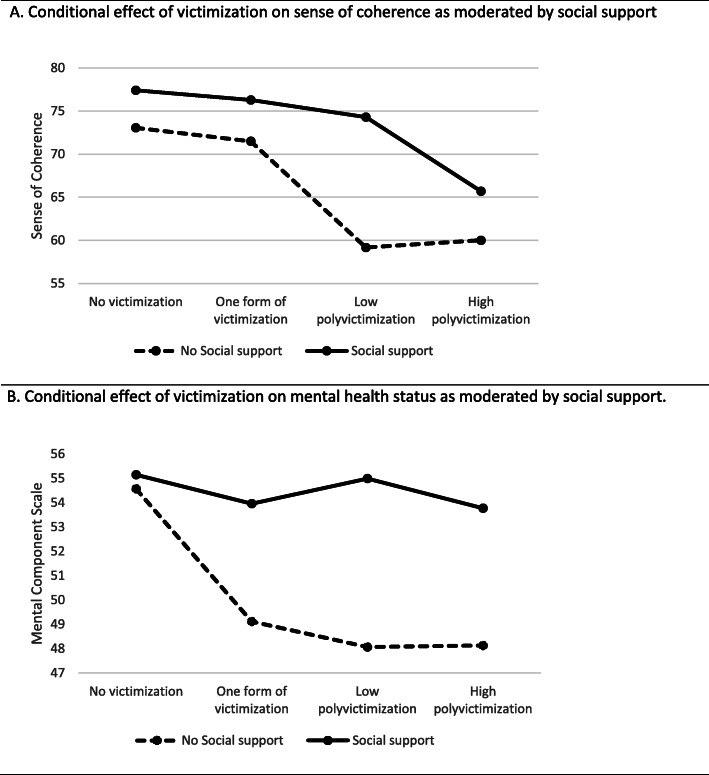
Visualization of moderating effects. **a** Conditional effect of victimization on sense of coherence as moderated by social support. **b** Conditional effect of victimization on mental health status as moderated by social support

In the conditional process analysis, a significant moderated (social support) mediation (sense of coherence) was found for the association between polyvictimization and both mental and physical health status (Tables 4 and 5). There was a negative association between victimization and SOC, and as is visualized in Fig. 2a, the association was moderated by social support. Respondents reporting no social support had lower levels of SOC in all victimization categories, but the effect was most prominent in the category low polyvictimization (mean SOC score among victims with social support was 74.3 and for victims without social support mean SOC score was 59.2) (Fig. 2a). The index of moderated mediation for low polyvictimization was 3.98 (Bootstrap CI 0.09–8.39). As this index is separate from 0, a significant moderating mediation was found. The mediation model supports SOC as a resilience factor; we found a positive correlation between SOC and both MCS (b = 0.37, 95% CI 0.30–0.43, *p* <0.01) and PCS (b = 0.16, 95% CI 0.08–0.25, *p* <0.01). Hence, we found an overall negative association between polyvictimization and mental as well as physical health status, mediated by SOC, and moderated by social support. No effect was seen for reporting only one form of victimization (Tables 4 and 5).

**Table 4:** Final model for the association between violence victimization and mental health status. A moderated (social support) mediation (sense of coherence) effect was found for victimization on mental health status. Social support had a near significant moderation effect also on the direct effect of victimization on mental health status


		Effect	95% CI	
**Relative conditional indirect effect (ab)** **(Victimization – SOC – Mental health status, moderated by social support)**				
**With social support**	One form of victimization	−0.42	−1.62	0.76
	Low polyvictimization	**−1.14**	**−2.29**	**−0.21**
	High polyvictimization	**−4.32**	**− 7.17**	**−2.02**
**No social support**	One form of victimization	−0.58	−3.28	1.95
	Low polyvictimization	**−5.13**	**−9.75**	**−1.34**
	High polyvictimization	**−4.81**	**−7.59**	**−2.29**
Significance test for moderating effect of social support: Index of moderated mediation for low polyvictimization: 3.98; CI 0.09–8.39				
**Relative conditional direct effect (c’)** **(Victimization - Mental health status, moderated by social support)**				
**With social support**	One form of victimization	−1.16	−3.83	1.51
	Low polyvictimization	0.08	−2.12	2.28
	High polyvictimization	−1.65	−5.09	1.79
**No social support**	One form of victimization	**−5.38**	**−10.02**	**−0.73**
	Low polyvictimization	**−6.96**	**−12.79**	**−1.12**
	High polyvictimization	**−6.19**	**−10.75**	**−1.63**
Significance test for moderating effect of social support: Test of highest order unconditional interaction: *p* = 0.06 Test of equality means: with social support *p* = 0.67, no social support *p* = <0.01				

Note: Model summary R^2^ = 0.36. Included in analyses = 471, Missing cases = 136. Confidence intervals are computed using bootstrapping with 5000 sample. No moderating effect of SOC was found (interaction effect *p* = 0.53)

**Table 5:** Final model for the association between violence victimization and physical health status. Sense of coherence (SOC) was found to have a mediating effect on the association between polyvictimization and physical ill-health. No significant effect was found for reporting only one form of victimization. Social support moderated the mediation effect but not the direct effect


		Effect	95% CI	
**Relative conditional indirect effect (ab)** **(Victimization – SOC– Physical health status, moderated by social support)**				
**With social support**	One form of victimization	−0.25	−1.58	0.87
	Low polyvictimization	**−2.26**	**−4.79**	**−0.49**
	High polyvictimization	**−2.12**	**−4.03**	**−0.73**
**No social support**	One form of victimization	−0.18	−0.83	0.30
	Low polyvictimization	**−0.47**	**−1.15**	**−0.02**
	High polyvictimization	**−1.90**	**−3.52**	**−0.64**
*Significance test for moderating effect of social support:* *Index of moderated mediation for low polyvictimization: 1.79; CI 0.06–4.07*				
**Relative direct effect (c’): Victimization - Physical health status** **(no significant moderation effect of social support)**				
One form of victimization		0.49	−2.59	3.57
Low polyvictimization		**−3.58**	**−6.35**	**−0.81**
High polyvictimization		**−3.83**	**−7.48**	**−0.17**

Note: Model summary R^2^ = 0.19. included in analyses *n* = 468, missing cases *n* = 139 Confidence intervals are computed using bootstrapping with 5000 sample. No moderating effect of SOC was found (interaction effect *p* = 0.81)

A near significant moderating effect of social support was found for the direct effect of victimization on mental health status (*p* = 0.06). The effect is visualized in Fig. 2b. A negative direct effect of victimization on mental ill-health was found only for those reporting no social support (one form of victimization − 5.38 95% CI: − 10.02; − 0.73, low victimization − 6.96 95% CI -12.79; − 1.12, and high polyvictimization − 6.19 95% CI -10.75; − 1.63).

No moderating effect of social support was found for the direct effect of victimization on physical health status. Therefore, a new model was constructed, eliminating social support as a potential moderating variable for the direct effect. In the new model, a relative direct effect was found: low polyvictimization (− 3.58, 95% CI -6.36; − 0.81), as well as high polyvictimization (− 3.83, 95% CI -7.48; − 0.17), but not reporting one form of victimization (0.49, 95% CI -2.59; − 3.57) was associated with a lower score on the PCS (Table 5).

## Discussion

Lifetime experiences of victimization were reported by every fourth man and woman in this study; among victims, polyvictimization was the norm rather than the exception. Lack of social support was the only background characteristic found to be associated with reporting high polyvictimization. Both resilience factors, SOC and social support, were found to affect the association between victimization and ill-health, indicating a possible target point for future interventions.

### Lifetime prevalence of violence victimization

It is generally difficult to compare the prevalence estimates of violence across studies due to different instruments and methodologies used. Our choice to study lifetime victimization makes comparisons even more difficult, considering that most studies concerning victimization among older adults only take into account victimization during the latest 12 months or since the age of 60 or 65. Previously, NorAQ has been found to report lower prevalence rates for intimate partner violence than other instruments [44]. Hence, it is more likely that our reported prevalence rates underestimate rather than overestimate the lifetime prevalence of victimization. Prevalence rates found in this sample are generally lower compared to our own previous studies of lifetime victimization in the general Swedish population, using the same instrument but younger samples [3, 44, 54]. Likewise, the Swedish National Council for Crime Prevention report generally lower prevalence rates of crimes, and especially violent crimes against older adults compared to younger adults [55]. A previous Swedish general population study also reports mostly lower prevalence rates among older respondents than younger [27]. The reasons for lower lifetime prevalence rates among older adults may reflect methodological issues. Recall bias concerning violent events occurring in youths is likely to be higher for older adults than for younger. Also, societal changes may be reflected in studies. In Sweden, corporal punishment of children was criminalized in 1979 and spousal rape was criminalized in 1965. In addition to laws, norms and values have shifted in society during the respondents’ lifetime. Also, women’s rights as well as children’s rights have come to the fore during the last decades. Hence, the respondents in this study have lived lives where violence against them was legal and normalized, which may have led to a lower likelihood of reporting their experiences as violent in this study. In accordance with this: we recently validated a screening instrument that can be used in health care to identify older patients with experiences of elder abuse or other life-time experiences of abuse [56]. In the validation process we found that some respondents had experienced violence earlier in life e.g., physical punishments in childhood, that they did not themselves considered to be abusive, but rather normative of that time [56].

We found that polyvictimization was the norm rather than the exception among victims. This may in part reflect a recall bias, i.e., respondents primarily report violence that was reoccurring while forgetting when only exposed to only one form of violence. Another reason for the high rate of polyvictimization could be the lifetime perspective, i.e., as time goes by experiences of victimization are likely to accumulate. However, three out of four respondents did not report any victimization. Hence, the high rate of polyvictimization is likely not due to a general overreporting of victimization, but rather that overall victimization is associated with an increased risk of multiple victimization. This interpretation is supported by previous findings that prior victimization is a strong risk factor for renewed victimization [3, 4, 6–8, 41].

Slightly more men (27.6%) than women (24.8%) reported some form of lifetime victimization in this study. It should however be underlined that the type of victimization was different between the sexes. More than twice as many female (62.7%) as male (25.8%) victims reported experiences of more than one form of violence (e.g., physical, emotional, and sexual) and almost twice as many female (40.9%) as male (23.6%) victims reported violence by more than one kind of perpetrator (partner, family member, other). Hence, women were more likely than men to report multiple forms of violence and violence from multiple perpetrators. Also, three times as many women (12.2%) as men (4.2%) reported violence by partners while more men (23.1%) than women (15.9%) reported “other” as the perpetrator (Table 2). This follows a well-known gendered pattern for victimization in younger ages and is consistent with a previous study among older adults in Sweden, where women were found to report more sexual violence and violence from intimate partners than were men [23].

In the multinomial regression model, the odds for reporting high polyvictimization was almost five times higher for respondents without social support compared to those with social support. This is consistent with previous findings that low social support is a risk factor for elder abuse [23, 40, 41] and also for elder polyvictimization [19].

### Victimization, ill-health, and resilience

As hypothesized, we found a negative association between victimization and both physical and mental health status. The association was most prominent for polyvictims; in fact, reporting only one form of victimization was not associated with physical health status at all and only had a direct association with mental health status among those that reported no social support (Tables 4 and 5). This confirms the importance of polyvictimization for the health of older adults.

Also as hypothesized, the association between victimization and health status was mediated by SOC. Consequently, victimization was associated with a lower SOC, and respondents with a lower SOC in turn reported a lower health status. A similar mediating effect of SOC on the relationship between violence at the workplace and stress reactions as well as vitality and mental health has previously been found [37]. When polyvictimization was first introduced as a concept, it was proposed that violence should not be understood as isolated events; rather, polyvictims were considered to live in a ‘violent condition’ [6]. Repeat victimization may influence victims’ strategies for coping in the aftermath of trauma and effect how they respond to subsequent victimization [57]. As visualized in Fig. 2a, we found a differentiated association between victimization and SOC; polyvictims had a lower SOC than those reporting only one form of violence and the lowest SOC was found for polyvictims with no social support. Likewise, when recently validating a new Swedish screening tool for elder abuse intended for use in healthcare, the REAGERA-S, an important finding was that experiences of violence earlier in life could still have a substantial negative impact on participants [56].

Social support was found to moderate the association between victimization and SOC. Victims reporting lack of social support also reported a lower SOC, and the difference is especially prominent for those reporting low polyvictimization (Fig. 2). This implies that social support may buffer the effect on SOC of being exposed to one form of violence and low polyvictimization, but for high polyvictims, social support cannot counteract the negative effect of victimization.

Victimization also had a direct effect on health status, i.e., not mediated by SOC. For mental health, this effect was moderated by social support at very close to significant levels (*p* = 0.06) (Table 4). Social support seems to buffer the effect of victimization on mental health, which is consistent with previous findings that social support moderates the association between depression and elder abuse [43]. Likewise, concerning intimate partner violence against younger women, a higher social support score has been associated with a reduced risk of poor health outcomes [58, 59]. However, we found no moderating effect of social support on the direct effect of victimization on physical health. Important aspects of social support reported are that social support helps the respondents to gain purpose in life as well as self-esteem and a sense of control or mastery of life [42]. Our measurement of social support consists of one item and does not necessarily cover these aspects. However, they are likely at least partly included in our measure of SOC, possibly explaining why we saw no moderating effect of social support on physical health but a mediating effect of SOC.

### Limitations

Ideally when studying the effect of lifetime polyvictimization, the study should include all forms of violence across the lifespan i.e., include, but not be limited to, experiences of childhood abuse, intimate partner violence, sexual violence, online abuse, workplace bullying and elder abuse. Moreover, it should also consider important traumatic events such as institutional betrayal and specific types of violence, e.g., racist or homophobic violence and violence toward indigenous people. It is likely that many of these forms of violence are included in the present study but they cannot be clearly differentiated. This is because NorAQ includes questions pertaining to acts of violence and does not specify the context or age at victimization. It should especially be noted that although we studied older adults’ experience of victimization, two of the most frequent types of elder abuse is not included in this study, i.e., neglect and financial abuse. Hence, our prevalence rates cannot be considered to reflect the true burden of victimization among older adults. Also, we did not include any measure of timing of victimization, i.e., we do not know when victims were exposed to violence. It may very well be that the effect of victimization on ill-health may be different depending on if violence occurred recently or a long time ago. However, previous findings suggest that even violence occurring many years ago may have a substantial impact on older adults [16, 56].

In studies concerning elder abuse, generally, only violence in relationships where there is a societal expectation of trust is included. Our choice to include also “other” as a potential perpetrator was based on previous findings that reporting violence from acquaintances and/or strangers is associated with an increased odds of also reporting victimization by family members and/or partners [4]. Likewise, reporting violence by acquaintances and/or strangers, in addition to violence by an intimate partner or family member, has been found to have an aggravated effect on the association between victimization and ill health [3].

Another limitation is that this study is a cross-sectional study; hence, we measured statistical association and mediation, not causality. We inquired about lifetime experiences of violence, and our health measure concerns health status and health related well-being during the last 4 weeks. Thus, it is plausible that our proposed direction of association is true, i.e., victimization leads to ill-health. However, different forms of ill-health are well-known risk factors for elder abuse [41, 60, 61]. Accordingly, it is possible that the direction of associations is reversed, or perhaps most likely, bidirectional.

It has previously been stipulated that it is important to consider both frequency and type of victimization in studies [19]. One strength of our polyvictimization variable is that it considers both frequency and duration of victimization as well as different forms of violence and kinds of perpetrators. A weakness is that we only consider kinds of perpetrators, not number of perpetrators and that different severities of violence were given the same score as more mild forms. However, in studies of polyvictimization among children and youth, the most prominent risk factor for repeat victimization as well as negative health outcome has been found to be the total number of types of victimization rather than any specific form of violence [6, 9, 10].

Data was collected in 2012 and it cannot be ruled out that the results would be different if the study had been conducted today. The prevalence of victimization among respondents age 25–65 in the original sample (from which the current sample was drawn) have been compared to respondents in another data collection using the same instrument and the same general population, but conducted in the year 2000 (women) and 2007 (men) [44]. There were no differences in reported overall lifetime prevalence rates for each form of violence for neither sex. This is an indication that lifetime prevalence of victimization is rather stable over time and that our results are valid even though time has passed.

Generalizability of the study results should be interpreted considering all mentioned strengths and limitations. Though we used a random population sample and well validated instruments the different methodological choices made and previously discussed may have affected our results.

### Research and clinical implication

Victimization accumulates over a life-course, but people are not powerless to the negative effects of victimization. Both SOC and social support affected the association between victimization and ill-health. Though not conclusive, previous findings suggest that SOC can be promoted in interventions among older adults, especially when interventions are sustained over several months [62, 63]. SOC was also found to increase after group therapy for adult women who were sexually abused as children [64]. Taken together, this indicates that SOC may be a useful component in future interventions also for older adults subjected to violence. Similarly, the theory of cumulative inequality stipulates that although inequality accumulates over a lifetime, individuals respond; hence, human agency may modify the life trajectory [17]. Concepts similar to SOC, e.g., sense of purpose and recovering positive affect, have been found to correlate with positive health outcome after controlling for victimization and other adversities among adolescents and adults [31, 32]. Hence, how we respond to trauma is affected among other things by coping and social processes and these factors have therefor been stipulated to be a salient point for intervention strategies [16]. In a recent analysis using the entire population from which the current sample was drawn, we found that only one out of eight victims of violence had ever been asked questions about victimization in health care [65]. If victims of violence could be more readily identified in health care, proper support might be given to strengthen resilience factors and coping strategies and thereby minimizing both the risk for revictimization and ill-health in the aftermath of violence. Therefore, further research and a better understanding of factors that foster resilience and that can be targeted in interventions are warranted [2, 16, 32]. Interventions could have different goals, e.g., to help older adults recover in the aftermath of victimization or aim to reduce further victimization. How and when to best intervene are for future studies to decide.

## Conclusion

Polyvictimization is a part of the life trajectory for many older adults and has negative health effects for victims. However, adverse health outcomes after victimization are not inevitable; we found that both high SOC and social support protect against adverse health effects of victimization. Considering the well-known detrimental health effects of victimization, more efforts should be made in health care to identify and help older adults subjected to violence. Our finding suggests that one way forward could be to create interventions focusing on strengthening different forms of resilience among victims, e.g., social support and sense of coherence. However, more research is needed to better understand the resilience factors and to investigate how these can be supported. Especially longitudinal studies are warranted to better investigate how health and a life free from violence can be promoted.

## Data Availability

The dataset supporting the conclusions of this article is available in the Harvard Dataverse repository 10.7910/DVN/DUNBML

## Abbreviations

LR: Likelihood Ratio
MCS: Mental Component Summary subscale
NorAQ: NorVold Abuse Questionnaire
PCS: Physical Component Summary subscale
PTSD: Post Traumatic Stress Disorder
SF-12: Short Form Survey 12 items
SOC: Sense of Coherence
